# Diseases of the Dental Pulp

**Published:** 1883-07

**Authors:** C. F. W. Bodecker


### SIMPLIFIED TREATMENT OF DISEASES OF THE DENTAL PULP BY THE USE OF IODOFORM, CARTILAGE AND TERCHLORIDE OF PHENOL.

In the *Deutsche Monatrschrift fur Zahnheilkunde*, May, 1883, Otto Wahlkoff, of Berlin, publishes two preparations used for treating and capping pulps, and filling root canals. The advantages claimed for the first of the above named materials are, that: "It will be resorbed when in contact with living tissues; it is easily introduced into pulp canals ; it absorbs exudations ; becomes hard when mixed with certain chemical substances, but is destroyed by pus ; is a very bad conductor of heat, and absolutely non-irritant." The material is prepared from ivory, hippopotamus, or bone shavings, or filings, decalcified in a ten per cent solution of chemically pure hydrochloric acid. After all the lime salts are extracted the residue is collected upon a filter, washed, dried and rubbed to a fine powder in a porcelain or glass mortar. Upon this powder ten times its weight of a ten per cent solution of iodoform in sulphuric ether is gradually poured, and constantly rubbed until a fine yellow powder is obtained, which contains about fifty per cent of decalcified bone, and fifty per cent of iodoform.

When used for capping pulps or filling roots this powder is made into a paste by the addition of carbolic acid or ter-chloride of phenol, rubbed together about five minutes, like ordinary cement.

Ter-chloride of phenol was introduced by Dianin of Russia, about a year ago, as the best disinfectant in gangrenous ulcers, "It is prepared by letting a stream of chlorine gas pass through chemically pure carbolic acid, previously melted, until it acquires a violet hue."

"This preparation," Dr. Wahlkoff says, "is not an irritant, has no acid reaction, and does not destroy the enamel of the teeth." Besides this he recommends the preparation as an obtundent for sensitive exposed necks of teeth, being careful, however, to polish the dentine after the application.

The author claims that with these two remedies he is prepared to meet almost every case of pulp disease. He classifies these diseases in four groups. In chapter one Wahlkoff describes the hyperæmic conditions of the pulp which he treats by applying a cap of iodoform cartilage mixed with ter-chloride of phenol.

Chapter two describes inflamed pulps exposed through caries; here the writer recommends the application of ter-chloride of prenol, repeated every third or fifth day until the patient experiences no more pain, and the pulp, if visible, has a normal appearance. The pulp is then to be capped with iodoform cartilage mixed with ter-chloride of phenol, and the cavity temporarily filled. Wahlkoff states that in twenty cases of pulpitis treated in this manner he only met with one failure.

The treatment of ulceration and gangrene of the pulp consists in the complete removal of all pulp tissues if possible, applications of ter-chloride of phenol into the root canals, and in the sealing of the cavity after the second or third application. Then, if no more trouble arises, the root canals are filled with iodoform cartilage mixed with ter-chloride of phenol.

The instruments recommended for the introduction of the iodoform cartilage, are of whalebone, which every one can make for himself.

According to the author, the preparation of the ter-chloride of phenol would appear to be a very simple matter, but in reality it is well known by chemists, that when carbolic acid is acted upon by chlorine gas, a number of compounds is the result. There are formed a mono, a bi, and a tri or ter-chloride of carbolic acid (phenol), the preparation of which, as well as their individual tests, will give no end of trouble to an amateur chemist. Furthermore, there is not one, but three different compounds of each of these chlorides. From the paper of Wahlkoff it does not appear which of these agents he has employed. I myself am not sufficiently competent, nor do I wish to pass judgment in matters purely chemical, but I had to refer to these difficulties, inasmuch as I would cordially invite the dental profession to give both the terchloride of phenol, as well as the so-called iodoform cartilage a trial. For it would seem, both from the results of Wahlkoff, as well as the authorities he cites (Sauer, Zimmerman, Barbe, Blume, Koser and Richter), that their properties are valuable. As regards the former, we would leave the preparation to the chemist, and then it would become necessary to fix upon the compound of the chloride of phenol which Wahlkoff has used, and which we are to employ hereafter. It is hardly necessary to say that iodoform *cartilage* is a misnomer for simply decalcified dead bone; the term has, however, the merit of shortness.

C. F. W. Bodecker.

